# Scalable fabrication of printed Zn//MnO_2_ planar micro-batteries with high volumetric energy density and exceptional safety

**DOI:** 10.1093/nsr/nwz070

**Published:** 2019-06-11

**Authors:** Xiao Wang, Shuanghao Zheng, Feng Zhou, Jieqiong Qin, Xiaoyu Shi, Sen Wang, Chenglin Sun, Xinhe Bao, Zhong-Shuai Wu

**Affiliations:** 1 Dalian National Laboratory for Clean Energy, Dalian Institute of Chemical Physics, Chinese Academy of Sciences, Dalian 116023, China; 2 University of Chinese Academy of Sciences, Beijing 100049, China; 3 State Key Laboratory of Catalysis, Dalian Institute of Chemical Physics, Chinese Academy of Sciences, Dalian 116023, China; 4 Department of Chemical Physics, University of Science and Technology of China, Hefei 230026, China

**Keywords:** low cost, printed, planar, Zn//MnO_2_ micro-batteries, metal-free current collectors

## Abstract

The rapid development of printed and microscale electronics imminently requires compatible micro-batteries (MBs) with high performance, applicable scalability, and exceptional safety, but faces great challenges from the ever-reported stacked geometry. Herein the first printed planar prototype of aqueous-based, high-safety Zn//MnO_2_ MBs, with outstanding performance, aesthetic diversity, flexibility and modularization, is demonstrated, based on interdigital patterns of Zn ink as anode and MnO_2_ ink as cathode, with high-conducting graphene ink as a metal-free current collector, fabricated by an industrially scalable screen-printing technique. The planar separator-free Zn//MnO_2_ MBs, tested in neutral aqueous electrolyte, deliver a high volumetric capacity of 19.3 mAh/cm^3^ (corresponding to 393 mAh/g) at 7.5 mA/cm^3^, and notable volumetric energy density of 17.3 mWh/cm^3^, outperforming lithium thin-film batteries (≤10 mWh/cm^3^). Furthermore, our Zn//MnO_2_ MBs present long-term cyclability having a high capacity retention of 83.9% after 1300 cycles at 5 C, which is superior to stacked Zn//MnO_2_ batteries previously reported. Also, Zn//MnO_2_ planar MBs exhibit exceptional flexibility without observable capacity decay under serious deformation, and remarkably serial and parallel integration of constructing bipolar cells with high voltage and capacity output. Therefore, low-cost, environmentally benign Zn//MnO_2_ MBs with in-plane geometry possess huge potential as high-energy, safe, scalable and flexible microscale power sources for direction integration with printed electronics.

## INTRODUCTION

The emerging smart printed electronics with the integrated features of exceptional flexibility, thinness, light weight, and miniaturization have significantly inspired the relentless pursuit of low-cost, safe and environmentally benign printed microscale energy-storage devices with high performance [1–5]. Lithium thin-film micro-batteries (MBs) with energy density of 10 mWh/cm^3^ are the most popular microscale power sources for various microsystems. However, most reported MBs are usually constructed in a non-planar stacked geometry, resulting in bulky volume, limited flexibility, and inconvenient serial and parallel connection via metal interconnects. Also, such MBs are generally fabricated by complicated manufacture processes, e.g. the photolithographic technique, and present unsatisfactory safety issues with flammable organic electrolytes. To overcome this, aqueous-based printed MBs with a separator-free planar geometry is acknowledged as a highly competitive class of microscale power sources due to the intrinsic non-flammability, high ionic conductivity of aqueous electrolytes [5,6], and great advances of planar device geometry with extremely short ion-diffusion pathways [7,8]. It is noteworthy that the printed planar MBs are highly favorable for direct integration of printed electronics on a single substrate, simultaneously combining the characteristics of outstanding flexibility, designable shapes, adjustable sizes, and space-saving connections.

So far, various printing techniques have been developed for fabricating traditional stacked batteries [9–12], such as lithium ion batteries by 3D printing [11], Zn–Ag batteries by inkjet printing [13], and Zn–air batteries by screen printing [14]. Also, great progress has been made with planar lithium thin-film MBs [15], lithium-ion MBs [16], Zn//Ag_2_O [3], Zn//LiMn_2_O_4_ [17], Zn//LiFePO_4_ [17], 3D MBs [18–20], and micro-supercapacitors [21–24] through the development of various micro-fabrication techniques, such as photolithography [25], electrodeposition [26], spraying [9,27], laser scribing [28], mask-assisted filtration [16], inkjet printing [10], roll-to-roll printing [29], and 3D printing [11,12]. In particular, screen printing can effectively control the precise pattern design with adjustable rheology of the inks, and is very promising for large-scale application [29]. Besides, screen printing is recognized as a cost-effective, easy-processing, and mass-production methodology for the fast construction of MBs, having precise control over the performance, flexibility, and integration with printed microelectronics. To address the cost effectiveness and safety issues, aqueous rechargeable Zn//MnO_2_ batteries, characterized by high abundance, low cost, non-toxicity and safety of both Zn and MnO_2_, as well as high output voltage of 0.9–1.8 V in aqueous electrolyte and high capacity of 820 mAh/g [30–32], are rising as one of the most compelling candidates [33–36]. Nevertheless, low-cost and scalable fabrication of aqueous-based Zn//MnO_2_ planar MBs with multiple innovative form factors of high performance, flexibility, and integration still remains challenging.

Herein we report a cost-effective and industrially applicable screen-printing strategy for fast and scalable production of rechargeable Zn//MnO_2_ planar MBs, featuring high performance, superior flexibility, scalable applicability, and high safety. The Zn//MnO_2_ planar MBs, free of separators, were manufactured by directly printing the zinc ink as the anode (thickness of 6.4 μm) and γ-MnO_2_ ink as the cathode (thickness of 9.8 μm), high-quality graphene ink as metal-free current collectors, working in environmentally benign neutral aqueous electrolytes of 2 M ZnSO_4_ and 0.5 M MnSO_4_. Benefiting from the suitable rheological properties of the inks and high electrical conductivity of microelectrodes (463 S/m for zinc anode, and 339 S/m for MnO_2_ cathode), the as-fabricated Zn//MnO_2_ MBs showed outstanding volumetric capacity of 19.3 mAh/cm^3^ at 7.5 mA/cm^3^ (393 mAh/g at 154 mA/g), high energy density of 17.4 mWh/cm^3^, long-term cycling stability (∼83.9% after 1300 times at 5 C), designable shape, extraordinary flexibility, outstanding serial and parallel modularization for boosting the capacity and voltage output. Therefore, taking such impressive performance into account, our Zn//MnO_2_ MBs fabricated with screen-printing technology could potentially meet the stringent requirements of high performance, environmental friendliness, low cost, easy scalability, and high safety for printed electronics [37].

## RESULTS AND DISCUSSION

**Figure 1.**
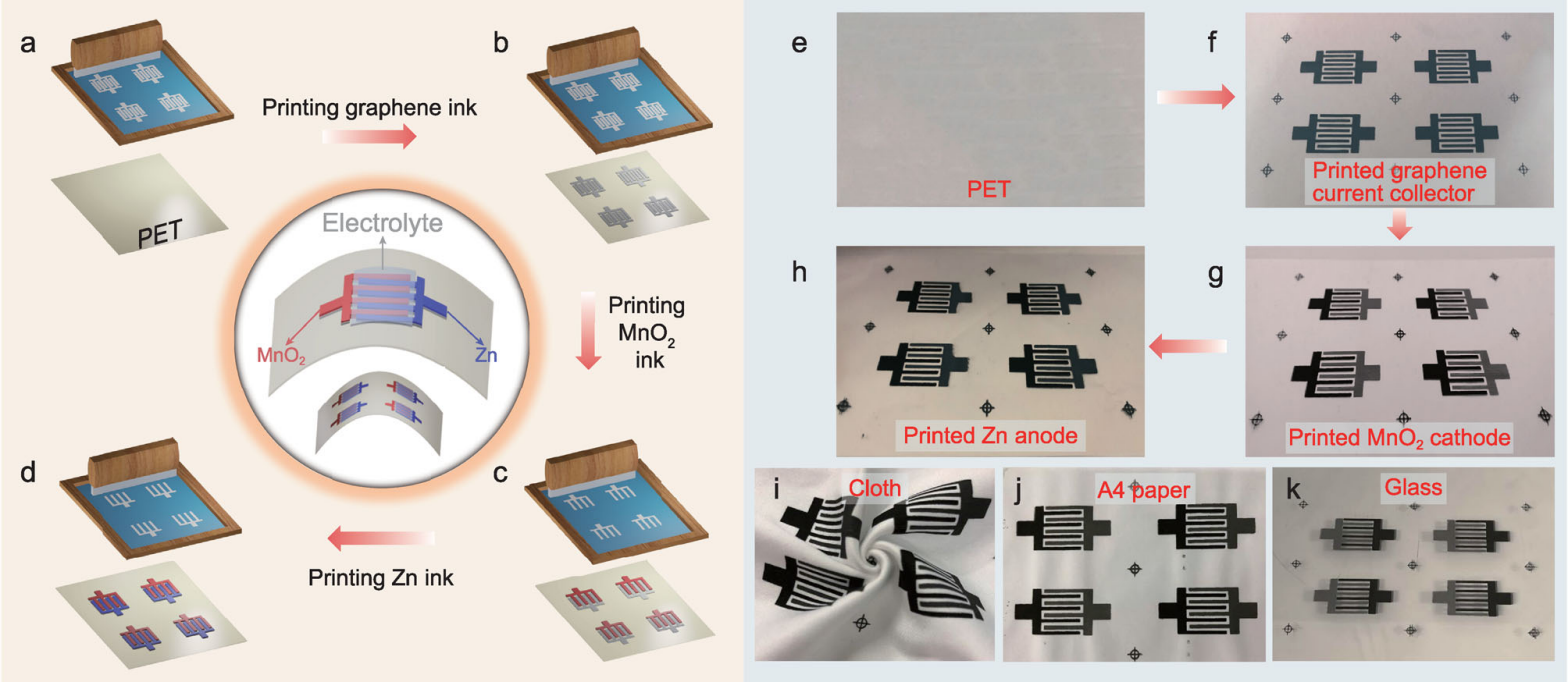
Fabrication of printed Zn//MnO_2_ planar MBs. (a–d) Schematic of screen-printing fabrication of printed Zn//MnO_2_ MBs: (a) the blank PET substrate, (b) the printed graphene current collectors, (c) the printed MnO_2_ cathode and (d) the printed Zn anode. (e–h) Optical photographs showing the stepwise printing fabrication of Zn//MnO_2_ MBs: (e) the blank PET substrate, (f) the graphene current collectors, (g) the printed MnO_2_ cathode and (h) the printed Zn anode on interdigital graphene fingers. (i–k) Zn//MnO_2_ MBs printed onto different substrates, including (i) cloth, (j) A4 paper, and (k) glass.

**Figure 2.**
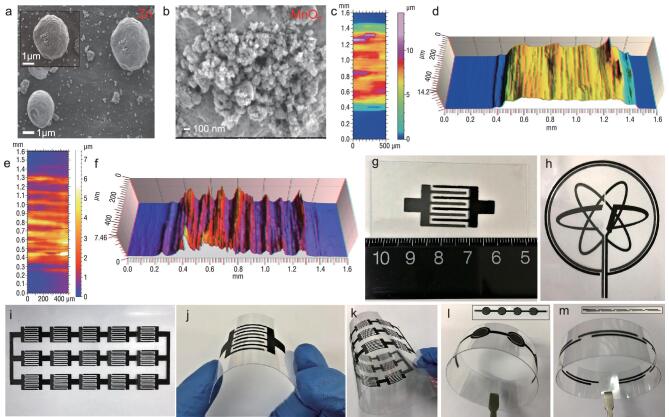
Characterization and shape diversity of printed Zn//MnO_2_ planar MBs: SEM images of (a) Zn anode and (b) MnO_2_ cathode; (c) 2D pseudo-color view and (d) 3D view of MnO_2_ microelectrode finger on PET substrate, showing the microelectrode thickness of ∼9.8 μm; (e) 2D pseudo-color view and (f) 3D view of Zn microelectrode finger on PET substrate, showing the microelectrode thickness of 6.4 μm; photographs of flexible Zn//MnO_2_ MBs with various shape diversity, including (g) individual interdigital structure, (h) ‘DICP’ logo-based Zn//MnO_2_ MBs, and (i) an energy-storage pack of Zn//MnO_2_ MBs connected in a tandem fashion of 5 series × 3 parallel; photographs of shape-designable Zn//MnO_2_ MBs under different bending states, including (j) an individual interdigital Zn//MnO_2_ MB, (k) tandem energy-storage packs via self-connection of (g) interdigital Zn//MnO_2_ MBs in 5 series × 3 parallel bent at 180°, (l) four concentric-circle-shape, and (m) five linear-shape Zn//MnO_2_ MBs in series, under flat and bending (180°) states.

The screen-printing fabrication of the interdigital Zn//MnO_2_ planar MBs is schematically illustrated in Fig. 1a–h. Firstly, highly stable and conductive graphene ink with appropriate rheological properties was printed on the substrates, e.g. flexible polyethylene terephthalate (PET), cloth, A4 paper, and even rigid glass (Fig. 1i–k), through a screen-printed process to form the interdigital planar patterns as metal-free current collectors, with a typical thickness of 1.4 μm, and exceptional electrical conductivity of 2.3 × 10^4^ S/m (Fig. S1a, Supporting Information). Secondly, the anodic four fingers of asymmetric interdigital microelectrodes were deposited by extruding Zn-based ink (33.3 wt% Zn microparticles) (Fig. 2a, Figs S2a and S3a, b, Supporting Information) through the screen on one side of the four graphene current collectors. Thirdly, the cathodic four fingers were manufactured by screen-printing γ-MnO_2_-based ink (18.8 wt% MnO_2_ nanoparticles) (Fig. 2b, Figs S2b, S3c, d, and S4, Supporting Information) on the other side of the four graphene-based current collectors. Notably, all inks possessed typical thixotropic behavior, showing a decreasing viscosity with increasing shear rate, staying below 1 Pa·s from 10 to 8000 s^−1^ (Fig. S5a–c, Supporting Information), which is highly important for precisely patterning microelectrodes [37,38]. The screen-printed Zn-based anode and MnO_2_-based cathode (SEM, Fig. S6, Supporting Information), with a typical thickness of 6.4 and 9.8 μm (Fig. 2c–f), exhibited high electrical conductivity of ∼320 and ∼450 S/m (Fig. S1b–c, Supporting Information), respectively. It is noted that the as-fabricated Zn//MnO_2_ planar MBs, free of both the separator and metal current collectors, exhibited extremely short ion-diffusion distances [39–42], and robust flexibility without film fracture and delamination from the substrate under various bending states (Fig. 2g–m) [8]. Furthermore, our screen-printing technique is highly simple, effective and scalable for low-cost production of flexible and seamlessly integrated Zn//MnO_2_ MBs with designable shapes and complex planar geometries, such as individual (Fig. 2g, j) and multiple parallel interdigital MBs via connection in series and in parallel (Fig. 2i, k), our institute logo MBs (Fig. 2h), tandem concentric circular (Fig. 2l) and linear MBs (Fig. 2m) not requiring conventional metal-based interconnectors. Finally, after adding the aqueous electrolyte (2 M ZnSO_4_ and 0.5 M MnSO_4_) onto the projected area of microelectrodes and packaging, aqueous-based Zn//MnO_2_ planar MBs were obtained.

**Figure 3.**
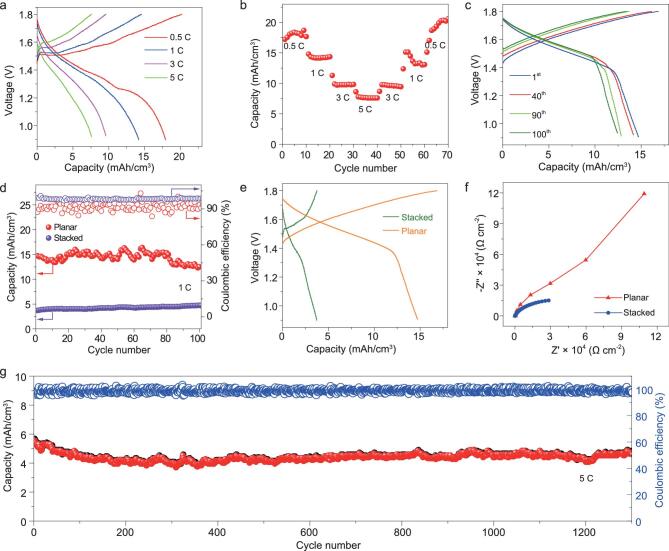
Electrochemical performance of printed Zn//MnO_2_ planar MBs: (a) the GCD profiles, and (b) rate capability of Zn//MnO_2_ MBs obtained from 0.5 C to 5 C; (c) the 1st, 40th, 90th, 100th GCD profiles, measured at a low rate of 1 C (15 mA/cm^3^); (d) cycling stabilities of printed Zn//MnO_2_ MBs with planar and sandwich-like stacked geometries, measured at a rate of 1 C; (e) the GCD profiles of the planar and stacked Zn//MnO_2_ MBs; (f) EIS normalized to 1 of the planar and stacked Zn//MnO_2_ MBs; (g) long-term cycling stability of Zn//MnO_2_ planar MBs over 1300 cycles at a high rate of 5 C.

To demonstrate the outstanding electrochemical performance, we first measured galvanostatic charge and discharge (GCD) profiles of printed Zn//MnO_2_ MBs at different current densities of 0.5 to 5 C (1 C = 308 mA/g, or 15 mA/cm^3^) between 0.9 and 1.8 V, using a neutral aqueous electrolyte containing 2 M ZnSO_4_ and 0.5 M MnSO_4_. It is pointed out that the presence of MnSO_4_ can significantly prevent the dissolution of MnO_2_ and improve the cyclability of Zn//MnO_2_ MBs [42]. As expected, the addition of 0.5 M MnSO_4_ into electrolyte indeed results in an impressively enhanced performance of Zn//MnO_2_ MBs (Fig. S7a, b, Supporting Information) [42]. Apparently, our Zn//MnO_2_ MBs displayed a similar discharge voltage plateau at ∼1.3 V observed at different current densities (Fig. 3a), originating from the intercalation mechanism in Zn//MnO_2_ MBs. Specifically, the insertion and extraction processes of both H^+^ and Zn^2+^ in the cathode are formulated as follows [43]:

$$\begin{equation*}\begin{array}{@{\hskip-32pt}l@{}} {{\rm{Cathode{:}\, Mn}}{{\rm{O}}_2} + {{\rm{H}}^ + } + {{\rm{e}}^ - } \leftrightarrow {\rm{MnOOH}}}\\
{{\rm{Z}}{{\rm{n}}^{2 + }} + 2{\rm{Mn}}{{\rm{O}}_2} + 2{{\rm{e}}^ - } \leftrightarrow {\rm{ZnM}}{{\rm{n}}_2}{{\rm{O}}_4}}\\
{{\rm{Anode{:}\,\,\,\,\, Zn}} \leftrightarrow {\rm{Z}}{{\rm{n}}^{2 + }} + 2{{\rm{e}}^ - }}
\end{array}\end{equation*}$$

Regardless of the increased rates, it was observed that the polarization did not virtually increase at high discharge rates. Importantly, our MBs presented exceptionally high capacity at the different rates. It was revealed that the discharge capacity varies from 18.4 (5th cycle), 14.2 (15th cycle), 9.8 (25th cycle) to 7.7 (35th cycle) mAh/cm^3^ with increasing rates from 0.5, 1, 3 to 5 C, respectively (Fig. 3b). Notably, the capacity thus readily returned to 9.6 (45th cycle), 13.6 (55th cycle) and 19.3 (65th cycle) mAh/cm^3^ when the rates go back to 3, 1, and 0.5 C, respectively (Fig. 3b).

The long-term cycling stability of Zn//MnO_2_ MBs is one of the most important performance metrics for actual applications. Through the elaborate screening of cathodic MnO_2_ and anodic zinc powder, selection of aqueous electrolytes (ZnSO_4_ + MnSO_4_), processing of highly stable and conducting inks, and usage of metal-free graphene current collectors, together with advanced planar geometry with a shorter ion-diffusion pathway and free of separator, and a sophisticated screen-printing technique, synergistically working together, the resulting Zn//MnO_2_ MBs showed remarkably satisfactory cycling performance (Fig. 3c, d, g). It is disclosed that the Zn//MnO_2_ planar MBs displayed an impressive capacity of 15 mAh/cm^3^ over 100 cycles at a low current density of 1 C. In sharp contrast, the stacked Zn//MnO_2_ MBs based on sandwich-like Zn foil and MnO_2_ electrode with a thickness of ∼200 μm, prepared by conventional blade coating (MnO_2_: acetylene black: polyvinylidene fluoride = 8:1:1), only showed about 4 mAh/cm^3^ after 100 cycles at 1 C (Fig. 3d), which definitely identify the superior performance of the Zn//MnO_2_ planar MBs. Importantly, the defined discharge voltage platform of the GCD profiles was still well maintained (Fig. 3e), further demonstrating the outstanding structural stability of microelectrodes. In addition, the GCD profiles show that the capacity of planar MBs (15 mAh/cm^3^) is much higher than stacked MBs (4 mAh/cm^3^). Electrochemical impedance spectroscopy (EIS) revealed that the slope of Zn//MnO_2_ planar MBs is much higher than the stacked cell at low frequencies (Fig. 3f, Fig. S8), indicative of the faster ion diffusion in the thinner microelectrodes of planar MBs. Furthermore, our Zn//MnO_2_ MBs displayed exceptional long-term cycling stability, with a capacity retention of 83.9% even at a high rate of 5 C after 1300 cycles (Fig. 3g), outperforming most reported Zn//MnO_2_ batteries, such as Zn//β-MnO_2_ (75% retention after 200 cycles) [30], Zn//MnO_2_@poly(3,4-ethylenedioxythiophene) (83.7% retention after 300 cycles) [33], yarn Zn//MnO_2_ (98.5% retention after 500 cycles) [43], and Zn//MnO_2_ (81.5% retention after 1000 cycles) [27]. The capacity decay was mainly attributed to the slow dissolution and disruption of the MnO_2_ cathode, the volume changes of microelectrodes owing to the large size of Zn^2+^ insertion/extraction, and the concomitant stresses on account of the irreversible side reaction [44]. Also, the capacity fluctuation of Zn//MnO_2_ MBs was mainly caused by the slow and non-uniform permeation of aqueous electrolyte into the electrodes during the cycling process. Several factors working together contributed to the outstanding electrochemical performance. First, metal-free graphene current collectors can significantly enhance the electrical conductivity of microelectrodes, and remarkably improve the rate capability of Zn//MnO_2_ MBs. Secondly, compared with α-MnO_2_ [45], the cathode of γ-MnO_2_ with the mixed tunnels of (1 × 1) and (1 × 2), is favorable for the Zn^2+^ ion intercalation/deintercalation in Zn//MnO_2_ MBs, and also highly active to the proton, following the so-called two-step pathways in a mild electrolyte. Thirdly, the aqueous electrolyte containing Zn^2+^ with a Mn^2+^ additive has a high ionic conductivity of >1.0 S/cm, three orders of magnitude higher than organic electrolytes (10^−3^ S/cm) [46,47], thereby greatly hindering the pulverization and dissolution of MnO_2_ and effectively improving the cyclability [48]. Last but not least, the polymer-assisted stable and conductive inks could substantially prevent the enormous volume change and the concomitant huge stress, thus contributing to the superior cyclability. As a result, the specific capacity and long-term cyclability of our Zn//MnO_2_ MBs are much better than those reported Zn//MnO_2_ batteries (Tables S1 and S2).

**Figure 4.**
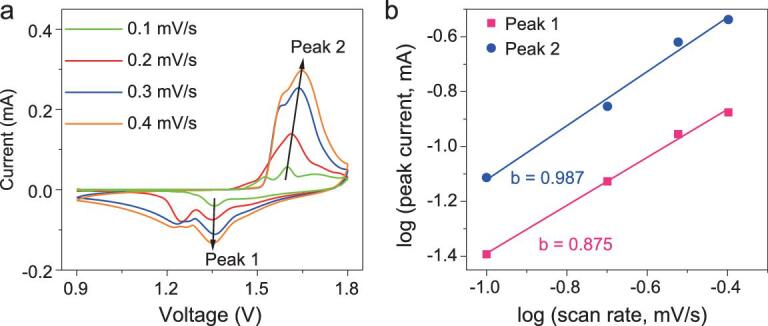
Kinetics analysis of ion intercalation of printed Zn//MnO_2_ planar MBs: (a) CV curves of Zn//MnO_2_ MBs obtained at various scan rates (*v*) from 0.1 to 0.4 mV/s; (b) plots of log (*i*) versus log (*v*) curves of cathodic and anodic peaks.

To properly understand the charge storage mechanism of Zn//MnO_2_ MBs, we further examined the cyclic voltammetry (CV) curves tested at different scan rates (Fig. 4a). It is evident that the two pair redox peaks (1.6 vs. 1.35 V, and 1.52 vs. 1.22 V) of the CV curves became gradually broader with increasing scan rate, but their shapes stayed almost consistent (Fig. 4a). To obtain an insight into the intrinsic mechanism of Zn//MnO_2_ MBs, we further analyzed the CV curves using the classic kinetics equations [49,50], *i* = *a v^b^* (or log *i* = log *a* + *b* log *v*), where the current *i* obeys a power-rule relationship with the scan rate *v*. Both *a* and *b* are adjustable parameters. The value *b* = 0.5 indicates a diffusion-controlled insertion process, while a *b* value of 1.0 represents a surface capacitive process. In terms of this regulation, it is calculated that, for our Zn//MnO_2_ MBs, the *b* values of four peaks are 0.875 (peak 1) and 0.987 (peak 2), respectively (Fig. 4b), indicating that the electrochemical kinetics of Zn//MnO_2_ MBs mainly involved the surface capacitive process, accompanied by the diffusion-controlled intercalation process to some extent, contributing to the superior performance [1].

**Figure 5.**
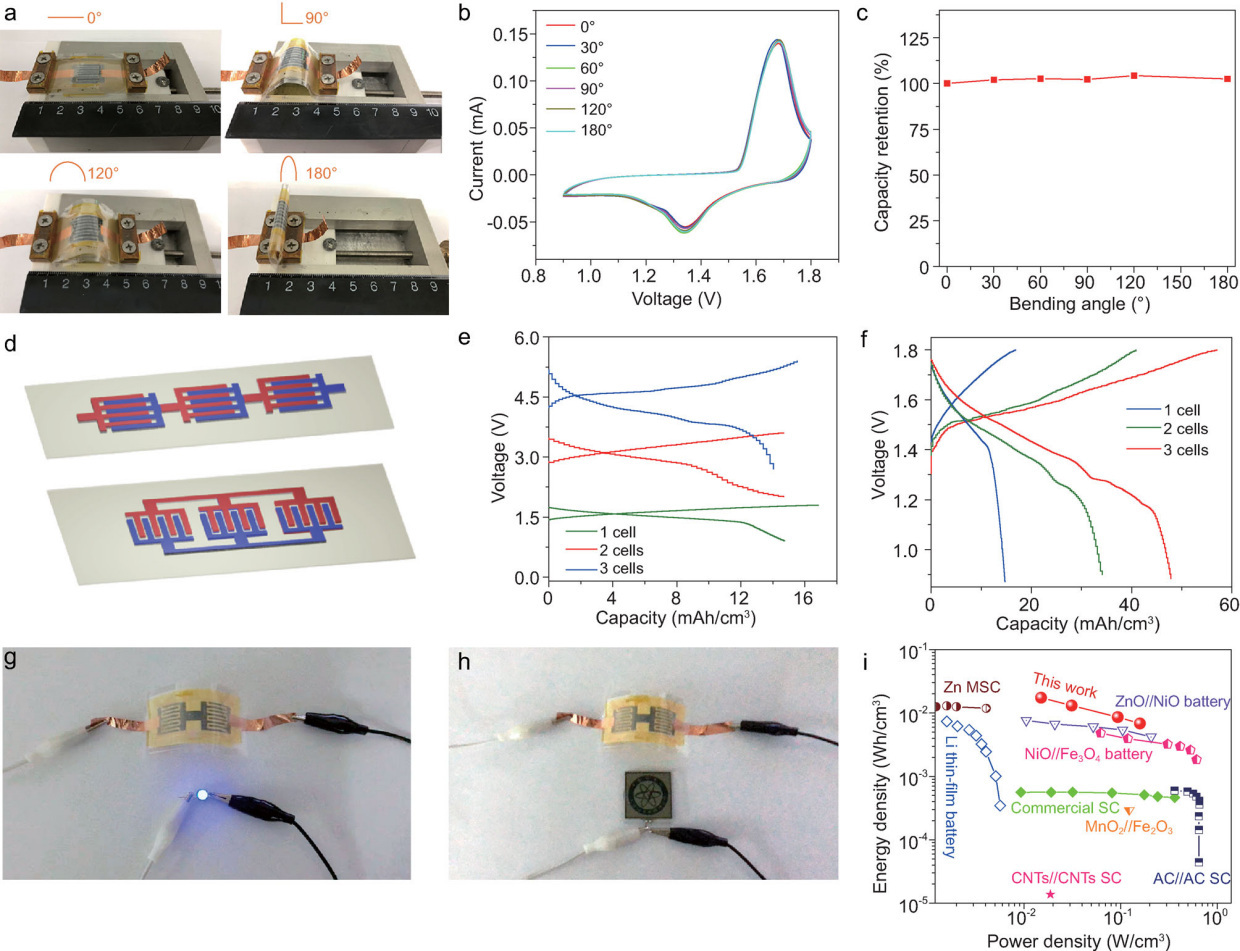
Exceptional flexibility and integration of Zn//MnO_2_ planar MBs. (a) Photographs, (b) CV curves, and (c) capacity retention of Zn//MnO_2_ MBs tested under different bending angles. (d) Schematic illustration of the integrated Zn//MnO_2_ MBs connecting three cells in series (top) and in parallel (bottom). (e, f) GCD profiles of the integrated Zn//MnO_2_ MBs connected in series (e) and in parallel (f) from 1 to 3 cells. (g, h) Photographs of two serially connected Zn//MnO_2_ MBs, lighting up a LED (g), and powering a display of our institute ‘DICP’ logo (h) in a flexible state. (i) Ragone plot of Zn//MnO_2_ MBs compared with other microscale energy-storage devices (AC: active carbon, CNTs: carbon nanotubes).

To meet the demands of future flexible and integrated microelectronics, developing flexible and integrated Zn//MnO_2_ MBs is urgently required. To highlight this feature, we examined CV curves of Zn//MnO_2_ MBs under varying bending angles from 0 to 180° at a scan rate of 1 mV/s (Fig. 5a). Apparently, it is confirmed that all the CV curves with typical battery behavior are well overlapped (Fig. 5b), along with an extraordinary capacity retention of almost 100% even when bent at 180^o^ (Fig. 5c), suggestive of highly stable flexibility. This is attributed to the advance of the separator-free planar geometry of Zn//MnO_2_ MBs built on one substrate, which can greatly enhance the intimate contact between the microelectrodes and flexible PET substrate, without involving the multiple interfacial delamination of stacked MBs [17].

Furthermore, the integrated Zn//MnO_2_ MBs were constructed via connection of multiple cells in series and in parallel (Fig. 5d), free of metal-based interconnects. It is worth noting that, from the GCD profiles, Zn//MnO_2_ MBs connected in series displayed analogical electrochemical properties, and simultaneously a stepwise increase of output voltage from 1.3 V for a single cell to 2.6 V for two cells and 3.9 V for three cells (Fig. 5e), suggestive of exceptional performance uniformity. Moreover, in a parallel fashion, the volumetric capacity of the integrated Zn//MnO_2_ MBs connected from one to three cells increased progressively, while the output voltage stayed almost unchanged (Fig. 5f). Notably, a tandem pack of two serially connected Zn//MnO_2_ MBs can readily power a light-emitting diode (LED) for a significantly long time under the flexible state, and light up a display screen of our institute ‘DICP’ logo, manifesting the enormous potential of our integrated Zn//MnO_2_ MBs (Fig. 5g, h).

The volumetric energy density and power density are also important performance metrics to evaluate microscale energy-storage devices; therefore, a Ragone plot is shown to compare our Zn//MnO_2_ planar MBs with other miniaturized energy-storage devices (Fig. 5i). Encouragingly, our printed Zn//MnO_2_ MBs could output a maximum volumetric energy density of 17.3 mWh/cm^3^ at a power density of 150 mW/cm^3^. This energy density is much higher than the commercially available supercapacitors (SC: 1 mWh/cm^3^), Zn-ion microsupercapacitors (Zn-MSC: 11.81 mWh/cm^3^) [2], and lithium thin-film battery (10 mWh/cm^3^) [51], ZnO//NiO (11 mWh/cm^3^) [52], and NiO//Fe_3_O_4_ (1.83 mWh/cm^3^) [53]. In addition, the power density of the Zn//MnO_2_ MBs is 150 mW/cm^3^, three orders of magnitude higher than a lithium thin-film battery (0.08 mW/cm^3^). Therefore, our printed Zn//MnO_2_ MBs not only manifest the merits of green, low-cost, scalable, and safe characteristics, but also possess high volumetric energy and power densities, making them suitable for numerous potential applications in miniaturized and printed electronics.

## CONCLUSIONS

In summary, we have demonstrated the cost-effective and scalable fabrication of rechargeable printed Zn//MnO_2_ planar MBs, with intriguing features of scalability, environmental benignity, high safety and metal-free current collectors, possessing high volumetric energy density, excellent rate capability and long-life cycling durability. Significantly, our printed Zn//MnO_2_ MBs could be designed with various planar configurations, simultaneously representing designable artistic shapes, impressive flexibility, and remarkable modularization of building bipolar cells with high voltage and capacity output. More importantly, taking into the full considerations of low-cost and safe Zn, earth-abundant MnO_2_, environmentally benign neutral aqueous electrolyte, and inexpensive screen-printing technology, our strategy of constructing printed Zn//MnO_2_ MBs holds great potential as next-generation microscale power sources in various wearable, flexible, miniaturized and printed electronics [18].

## METHODS

### Preparation of Zn ink and MnO_2_ ink

Polyurethane resin (99%, Henan DaKen Chemical Co., Ltd) was added into the dispersant of aromatic solvents (S150, 98%, Pengchen New Material Technology Co., Ltd) and ethylene glycol diglycidyl ether (99%, Hangzhou Dayangchem Co., Ltd). To fully dissolve the resin, the mixture was heated to 80°C for 2 h. Subsequently, graphene (Nanjing XFNANO Materials Tech Co., Ltd), superfine graphite (Wuxi Hengtai Metal Material Co., Ltd), carbon black (90%, Zhengzhou Blue Ribbon Industry Co., Ltd) and MnO_2_ powder (99.9%, Beijing DK Nano Technology Co. Ltd) were put into the above resin solution with an intense stirring of 1500 r/min for 30 min. After the resultant precursor was repeatedly ground under a three-roll grinder, MnO_2_ ink was achieved. The mass proportion of polyurethane resin: graphene: superfine graphite: carbon black: MnO_2_ powder is 3: 1: 1: 3: 2. The graphene conductive ink was prepared using the same reagent and procedure with graphene nanosheets (lateral size of 5–10 μm, 3–6 layers, Fig. S9), except with no addition of MnO_2_. The Zn ink was made by uniformly mixing the as-prepared conductive ink and zinc powder (6–9 μm, 97.5%, Alfa Aesar) with a weight ratio of 2:1, when it was used.

### Fabrication of Zn//MnO_2_ MBs

Firstly, the highly conductive graphene ink was first printed on the PET, A4 paper, glass, or cloth substrates to form graphene-based current collectors and dried at 80°C in a vacuum box for 20 min until totally dry. Secondly, Zn-based ink via the same approach was overlapped as anode on one side of the graphene-based current collectors, while MnO_2_-based ink was subsequently deposited as cathode on the other side of the graphene-based current collectors. Then, the screen-printed asymmetric microelectrodes of Zn//MnO_2_ MBs were dried at 80°C for 12 h. Afterwards, the neutral aqueous electrolyte of 2 M ZnSO_4_ and 0.5 M MnSO_4_ was slowly dropped onto the project area of the microelectrodes and packaged with Kapton tape. Finally, aqueous-based printed Zn//MnO_2_ MBs were obtained. Note that the interdigital customized screen has eight fingers, with length of 12 mm, width of 1 mm and interspace of 1 mm (Fig. S10).

### Materials characterization

The morphology, structure and composition of the active materials, graphene, the inks, and microelectrodes were analyzed using field-emission scanning electron microscopy (SEM, JSM-7800F), high-resolution transmission electron microscopy (HRTEM, JEM-2100), X-ray diffraction (XRD, X’pert Pro) (5–90°), four-point probe equipment (RTS-9), alpha step D-600, and thermogravimetric analysis (TGA, STA 449 F3) (measured at air atmosphere, 10°/min from 25 to 1000°C).

### Electrochemical measurement

The CV curves obtained at varying scan rates of 0.1–0.4 mV/s and EIS tested from 100 kHz to 0.01 Hz with an AC amplitude of 5 mV were conducted by an electrochemical workstation (CHI 760E), and the GCD profiles were measured by a LAND CT2001A battery tester at voltages between 0.9 and 1.8 V at current densities from 0.5 to 5 C.

## Supplementary Material

nwz070_Supplemental_File

## References

[1] Chao DL , Zhu C, Song M et al. A high-rate and stable quasi-solid-state zinc-ion battery with novel 2D layered zinc orthovanadate array. Adv Mater 2018; 30: 1803181. 10.1002/adma.201803181 29966034

[2] Sun GQ , Yang HS, Zhang GF et al. A capacity recoverable zinc-ion micro-supercapacitor. Energy Environ Sci 2018; 11: 3367–74.

[3] Kumar R , Shin J, Yin L et al. All-printed, stretchable Zn-Ag_2_O rechargeable battery via hyperelastic binder for self-powering wearable electronics. Adv Energy Mater 2017; 7: 1602096.

[4] Choi K-H , Yoo J, Lee CK et al. All-inkjet-printed, solid-state flexible supercapacitors on paper. Energy Environ Sci 2016; 9: 2812–21.

[5] Hondred JA , Stromberg LR, Mosher CL et al. High-resolution graphene films for electrochemical sensing via inkjet maskless lithography. ACS Nano 2017; 11: 9836–45. 28930433 10.1021/acsnano.7b03554

[6] Pan H , Shao Y, Yan P et al. Reversible aqueous zinc/manganese oxide energy storage from conversion reactions. Nat Energy 2016; 1: 16039.

[7] Wu ZS , Parvez K, Feng XL et al. Graphene-based in-plane micro-supercapacitors with high power and energy densities. Nat Commun 2013; 4: 2487. 24042088 10.1038/ncomms3487 PMC3778542

[8] Wu ZS , Feng XL, Cheng HM. Recent advances in graphene-based planar micro-supercapacitors for on-chip energy storage. Natl Sci Rev 2014; 1: 277–92.

[9] Singh N , Galande C, Miranda A et al. Paintable battery. Sci Rep 2012; 2: 5. 10.1038/srep00481 PMC3385420 22745900

[10] Deiner LJ , Reitz TL. Inkjet and aerosol jet printing of electrochemical devices for energy conversion and storage. Adv Energy Mater 2017; 19: 1600878.

[11] Sun K , Wei TS, Ahn BY et al. 3D printing of interdigitated Li-ion microbattery architectures. Adv Mater 2013; 25: 4539–43. 23776158 10.1002/adma.201301036

[12] McOwen DW , Xu SM, Gong YH et al. 3D-printing electrolytes for solid-state batteries. Adv Mater 2018; 30: 1707132. 10.1002/adma.201707132 29575234

[13] Ho CC , Murata K, Steingart DA et al. A super inkjet printed zinc−silver 3D microbattery. J Micromech Microeng 2009; 19: 094013.

[14] Hilder M , Winther-Jensen B, Clark NB. Paper-based, printed zinc−air battery. J Power Sources 2009; 194: 1135–41.

[15] Hu LB , Wu H, La Mantia F et al. Thin, flexible secondary Li-ion paper batteries. ACS Nano 2010; 4: 5843–8. 20836501 10.1021/nn1018158

[16] Zheng S , Wu Z-S, Zhou F et al. All-solid-state planar integrated lithium ion micro-batteries with extraordinary flexibility and high-temperature performance. Nano Energy 2018; 51: 613–20.

[17] Zhao JW , Sonigara KK, Li JJ et al. A smart flexible zinc battery with cooling recovery ability. Angew Chem Int Ed 2017; 56: 7871–5. 10.1002/anie.201704373 28503917

[18] Wei TS , Ahn BY, Grotto J et al. 3D printing of customized Li-ion batteries with thick electrodes. Adv Mater 2018; 30: 1703027. 10.1002/adma.201703027 29543991

[19] Oudenhoven JFM , Baggetto L, Notten PHL. All-solid-state lithium-ion microbatteries: a review of various three-dimensional concepts. Adv Energy Mater 2011; 1: 10–33.

[20] Janoschka T , Hager MD, Schubert US. Powering up the future: radical polymers for battery applications. Adv Mater 2012; 24: 6397–409. 23238940 10.1002/adma.201203119

[21] Wang S , Wu ZS, Zheng S et al. Scalable fabrication of photochemically reduced graphene-based monolithic micro-supercapacitors with superior energy and power densities. ACS Nano 2017; 11: 4283–91. 28350433 10.1021/acsnano.7b01390

[22] Xiao H , Wu Z-S, Zhou F et al. Stretchable tandem micro-supercapacitors with high voltage output and exceptional mechanical robustness. Energy Storage Mater 2018; 13: 233–40.

[23] Zheng S , Ma J, Wu Z-S et al. All-solid-state flexible planar lithium ion micro-capacitors. Energy Environ Sci 2018; 11: 2001–9.

[24] Zhou F , Huang H, Xiao C et al. Electrochemically scalable production of fluorine-modified graphene for flexible and high-energy ionogel-based microsupercapacitors. J Am Chem Soc 2018; 140: 8198–205. 29893575 10.1021/jacs.8b03235

[25] Wu ZS , Parvez K, Feng XL et al. Photolithographic fabrication of high-performance all-solid-state graphene-based planar micro-supercapacitors with different interdigital fingers. J Mater Chem A 2014; 2: 8288–93.

[26] Lai WH , Wang Y, Lei ZW et al. High performance, environmentally benign and integratable Zn//MnO_2_ microbatteries. J Mater Chem A 2018; 6: 3933–40.

[27] Shi XY , Wu ZS, Qin JQ et al. Graphene-based linear tandem micro-supercapacitors with metal-free current collectors and high-voltage output. Adv Mater 2017; 29: 1703034. 10.1002/adma.201703034 29028132

[28] Xie BH , Wang Y, Lai WH et al. Laser-processed graphene based micro-supercapacitors for ultrathin, rollable, compact and designable energy storage components. Nano Energy 2016; 26: 276–85.

[29] Choi KH , Ahn DB, Lee SY. Current status and challenges in printed batteries: toward form factor-free, monolithic integrated power sources. ACS Energy Lett 2018; 3: 220–36.

[30] Islam S , Alfaruqi MH, Mathew V et al. Facile synthesis and the exploration of the zinc storage mechanism of β-MnO_2_ nanorods with exposed (101) planes as a novel cathode material for high performance eco-friendly zinc-ion batteries. J Mater Chem A 2017; 5: 23299–309.

[31] Huang J , Wang Z, Hou M et al. Polyaniline-intercalated manganese dioxide nanolayers as a high-performance cathode material for an aqueous zinc-ion battery. Nat Commun 2018; 9: 2906. 30046036 10.1038/s41467-018-04949-4 PMC6060179

[32] Wang Z , Ruan Z, Liu Z et al. A flexible rechargeable zinc-ion wire-shaped battery with shape memory function. J Mater Chem A 2018; 6: 8549–57.

[33] Zeng YX , Zhang XY, Meng Y et al. Achieving ultrahigh energy density and long durability in a flexible rechargeable quasi-solid-state Zn-MnO_2_ battery. Adv Mater 2017; 29: 1700274. 10.1002/adma.201700274 28452147

[34] Zhang N , Cheng FY, Liu YC et al. Cation-deficient spinel ZnMn_2_O_4_ cathode in Zn(CF_3_SO_3_)_(2)_ electrolyte for rechargeable aqueous Zn-ion battery. J Am Chem Soc 2016; 138: 12894–901. 27627103 10.1021/jacs.6b05958

[35] Xia C , Guo J, Lei Y et al. Rechargeable aqueous zinc-ion battery based on porous framework zinc pyrovanadate intercalation cathode. Adv Mater 2018; 30: 1705580. 10.1002/adma.201705580 29226488

[36] Song M , Tan H, Chao D et al. Recent advances in Zn-ion batteries. Adv Funct Mater 2018; 28: 1802564.

[37] Wang ZQ , Winslow R, Madan D et al. Development of MnO_2_ cathode inks for flexographically printed rechargeable zinc-based battery. J Power Sources 2014; 268: 246–54.

[38] Gaikwad AM , Whiting GL, Steingart DA et al. Highly flexible, printed alkaline batteries based on mesh-embedded electrodes. Adv Mater 2011; 23: 3251–5. 21661062 10.1002/adma.201100894

[39] El-Kady MF , Kaner RB. Scalable fabrication of high-power graphene micro-supercapacitors for flexible and on-chip energy storage. Nat Commun 2013; 4: 9. 10.1038/ncomms2446 23403576

[40] Pech D , Brunet M, Durou H et al. Ultrahigh-power micrometre-sized supercapacitors based on onion-like carbon. Nat Nanotechnol 2010; 5: 651–4. 20711179 10.1038/nnano.2010.162

[41] Chmiola J , Largeot C, Taberna PL et al. Monolithic carbide-derived carbon films for micro-supercapacitors. Science 2010; 328: 480–3. 20413497 10.1126/science.1184126

[42] Huang J , Guo Z, Ma Y et al. Recent progress of rechargeable batteries using mild aqueous electrolytes. Small Methods 2019; 3: 1800272.

[43] Li H , Liu Z, Liang G et al. Waterproof and tailorable elastic rechargeable yarn zinc ion batteries by a cross-linked polyacrylamide electrolyte. ACS Nano 2018; 12: 3140–8. 29589438 10.1021/acsnano.7b09003

[44] Sun W , Wang F, Hou S et al. Zn/MnO_2_ battery chemistry with H^+^ and Zn^2+^ coinsertion. J Am Chem Soc 2017; 139: 9775–8. 28704997 10.1021/jacs.7b04471

[45] Wu B , Zhang G, Yan M et al. Graphene scroll-coated alpha-MnO_2_ nanowires as high-performance cathode materials for aqueous Zn-ion battery. Small 2018; 14: 1703850. 10.1002/smll.201703850 29392874

[46] Ding J , Du Z, Gu L et al. Ultrafast Zn^2+^ intercalation and deintercalation in vanadium dioxide. Adv Mater 2018; 30: 1800762. 10.1002/adma.201800762 29761561

[47] Konarov A , Voronina N, Jo JH et al. Present and future perspective on electrode materials for rechargeable zinc-ion batteries. ACS Energy Lett. 2018; 3: 2620–40.

[48] Fu Y , Wei Q, Zhang G et al. High-performance reversible aqueous Zn-ion battery based on porous MnO_x_ nanorods coated by MOF derived N-doped carbon. Adv Energy Mater 2018; 8: 1801445.

[49] Chao D , Zhu C, Yang P et al. Array of nanosheets render ultrafast and high-capacity Na-ion storage by tunable pseudocapacitance. Nat Commun 2016; 7: 12122. 27358085 10.1038/ncomms12122 PMC4931321

[50] Chao DL , Liang P, Chen Z et al. Pseudocapacitive Na-ion storage boosts high rate and areal capacity of self-branched 2D layered metal chalcogenide nanoarrays. ACS Nano 2016; 10: 10211–9. 27768284 10.1021/acsnano.6b05566

[51] El-Kady MF , Strong V, Dubin S et al. Laser scribing of high-performance and flexible graphene-based electrochemical capacitors. Science 2012; 335: 1326–30. 22422977 10.1126/science.1216744

[52] Liu J , Guan C, Zhou C et al. A flexible quasi-solid-state nickel–zinc battery with high energy and power densities based on 3D electrode design. Adv Mater 2016; 28: 8732–9. 27562134 10.1002/adma.201603038

[53] Zhao T , Zhang G, Zhou F et al. Toward tailorable Zn-ion textile batteries with high energy density and ultrafast capability: building high-performance textile electrode in 3D hierarchical branched design. Small 2018; 14: 1802320. 10.1002/smll.201802320 30106506

