# Supplementary material for: Scalable fabrication of printed Zn//MnO2 planar micro-batteries with high volumetric energy density and exceptional safety
Source: Natl Sci Rev. 2019 Jun 11;7(1):64–72. doi: 10.1093/nsr/nwz070 (PMC8288951; doi:10.1093/nsr/nwz070)
Supplement: nwz070_Supplemental_File [file nwz070_supplemental_file.docx]

**Supporting Information**

**Scalable Fabrication of Printed Zn//MnO_2_ Planar Micro-Batteries with High Volumetric Energy Density and Exceptional Safety**

*Xiao Wang^1,2^, Shuanghao Zheng^1,2,3^, Feng Zhou^1^, Jieqiong Qin^1,2^, Xiaoyu Shi^1,3,4^, Sen Wang^1^, ^2^, Chenglin Sun^1^, Xinhe Bao^1,3,4^, Zhong-Shuai Wu^1^,**

^1^ Dalian National Laboratory for Clean Energy, Dalian Institute of Chemical Physics, Chinese Academy of Sciences, 457 Zhongshan Road, Dalian 116023, P. R. China

^2^ University of Chinese Academy of Sciences, 19 A Yuquan Road, Shijingshan District, Beijing, 100049, P. R. China

^3^ State Key Laboratory of Catalysis, Dalian Institute of Chemical Physics, Chinese Academy of Sciences, 457 Zhongshan Road, Dalian 116023, P. R. China

^4^ Department of Chemical Physics, University of Science and Technology of China, 96 JinZhai Road, Hefei 230026, China.

*E-mail: wuzs@dicp.ac.cn (Z.-S.Wu)

**Calculations**

The specific volumetric capacity of Zn//MnO_2_ battery was calculated from the discharge curves using the following equation (1):

$C=\frac{It}{V_{device}}$ (1)

Where C is the volumetric capacity (mAh cm^-3^), *I* (mA) is the applied discharging current, *t* (h) is discharge time, $V_{device}$(cm^-3^) is the total volume of two active microelectrodes.

The volumetric energy density and power density of Zn//MnO_2_ batteries were evaluated by the formula (2) and (3):

$E=C\Delta V$ (2)

$P=\frac{E}{t}$ (3)

Where $\Delta V$ is the voltage window ($\Delta V$), *E* is volumetric energy density (Wh cm^-3^), *P* is volumetric power density (W cm^-3^).^[1]^


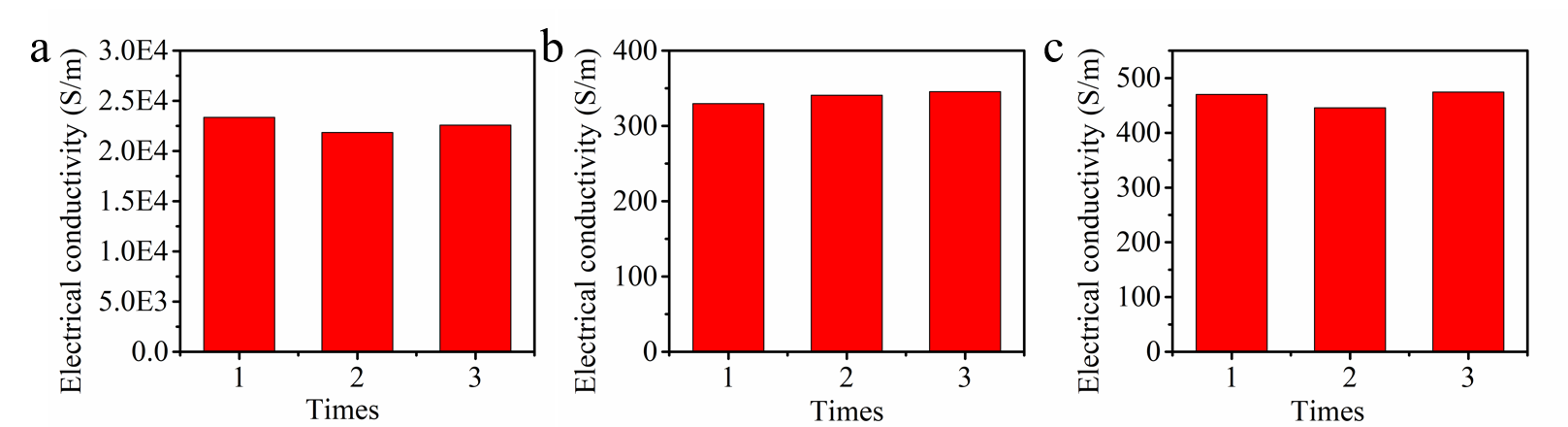


**Figure S1.** Electrical conductivity of printed microelectrode films using (a) graphene ink, (b) MnO_2_ ink, and (c) Zn ink on the PET substrates.


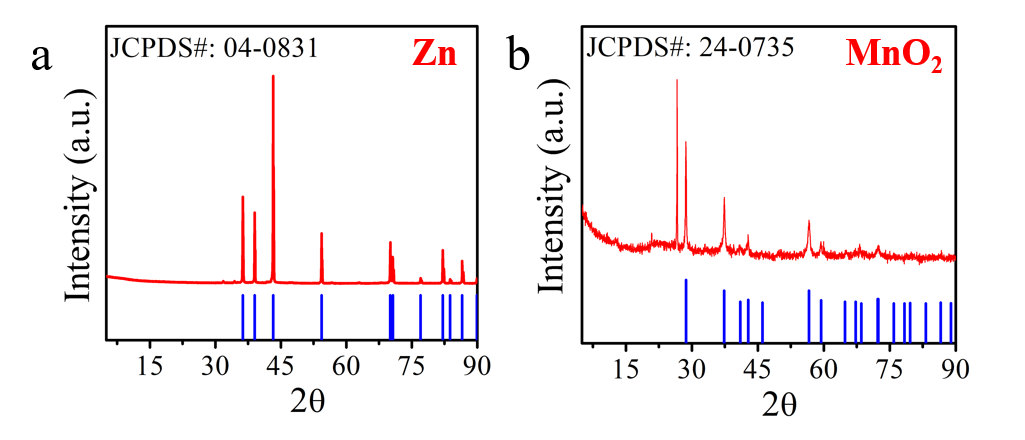


**Figure S2.** XRD patterns of (a) zinc powder and (b) MnO_2_ powder, showing the characteristic diffraction peaks of Zn and γ-MnO_2_.


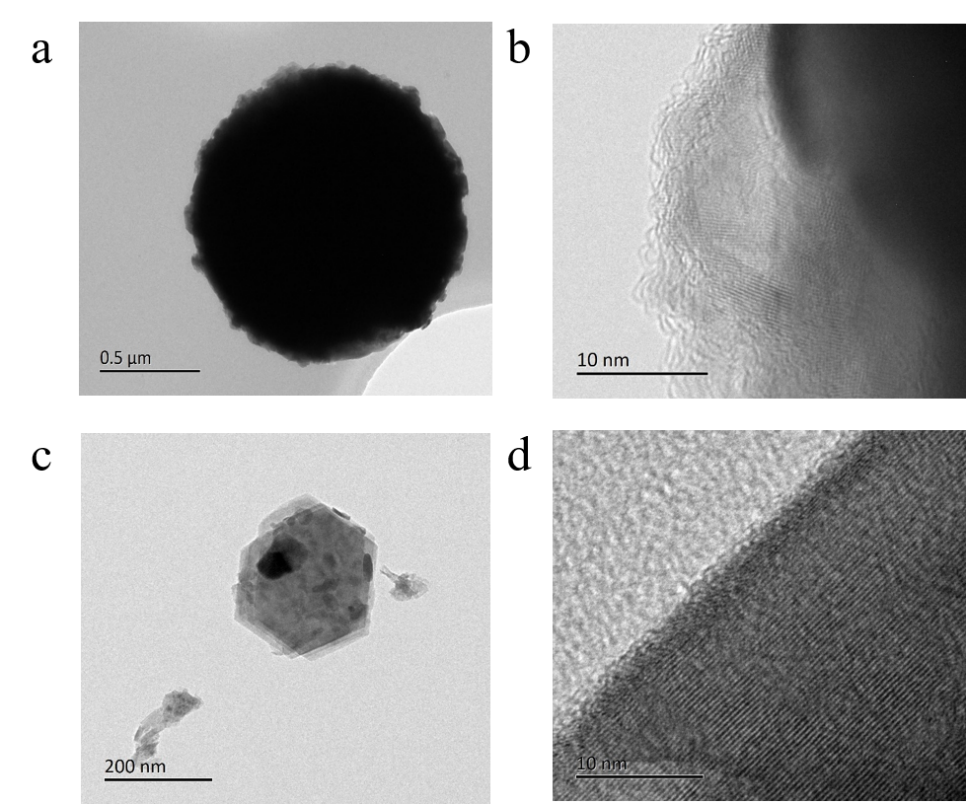


**Figure S3.** TEM images for (a,b) zinc powder and (c,d) MnO_2_ powder, showing the micrometer size of zinc (about 6~9 μm), and nanoparticles of MnO_2_ powder (about 50~100 nm).


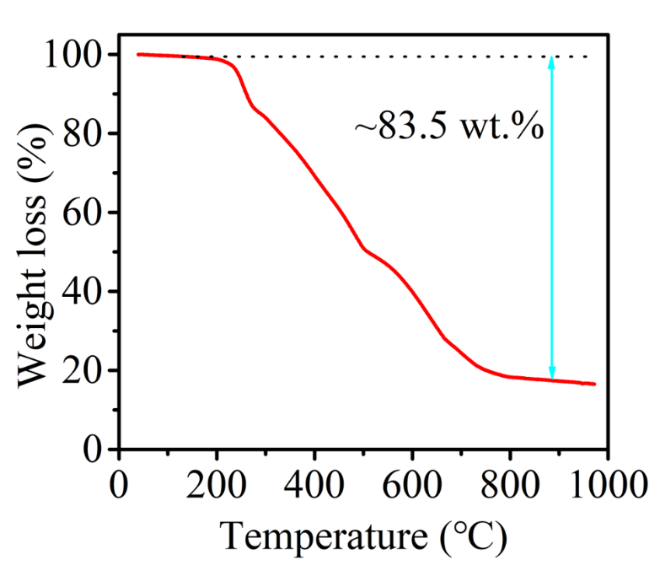


**Figure S4.** TG analysis of the MnO_2_ ink, from 25 to 1000 ^o^C at a heating speed of 10 ^o^C/min in the air.

Because of the morphology change of MnO_2_ heated at 1000 ^o^C, the MnO_2_ mass in the ink was calculated in term of the following equation: ^[2]^

3 MnO_2_ ↔Mn_3_O_4_ + O_2_

Since the residual of the ink was about 16.5% after 1000 ^o^C, it is calculated that the weight of MnO_2_ was about 18.8%.


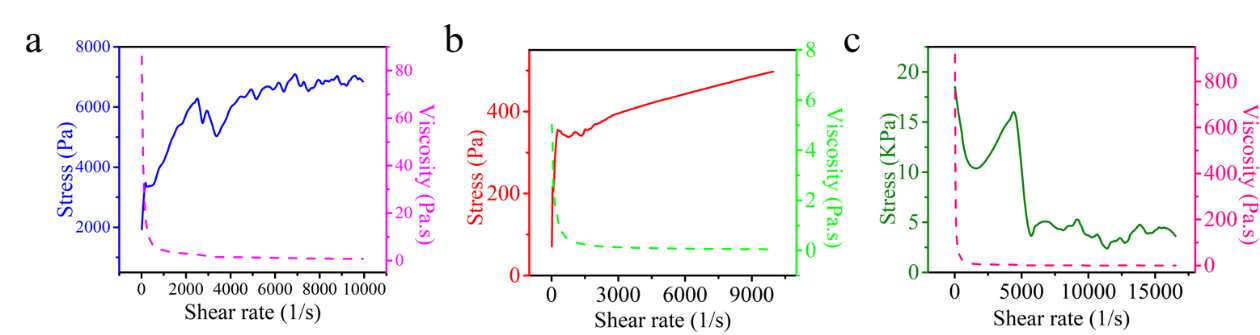


**Figure S5.** The rheological properties of (a) graphene ink, (b) MnO_2_ ink and (c) Zn ink. All ink viscosities keep below 1 Pa·s with increasing shear rate from 10 s^-1^ to 8000 s^-1^, exhibiting pseudoplastic behavior that is essential parameter for screen printing.^[3-4]^

**
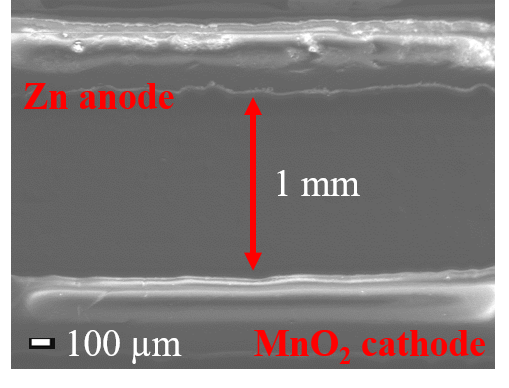
**

**Figure S6.** SEM image for microelectrodes of Zn/MnO_2_ MBs, with a thickness of 6.4 μm for Zn anode, and 9.8 μm for cathode


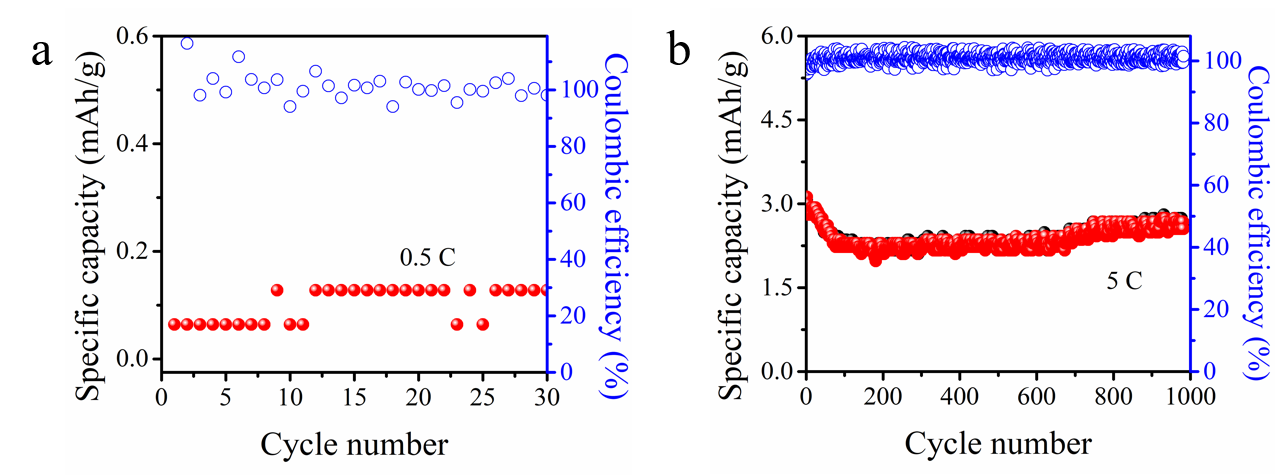


**Figure S7.** (a) Cycling stability of printed Zn/MnO_2_ MBs tested at a current density of 0.5 C without MnSO_4_. (b) Cycling performance of Zn/MnO_2_ MBs obtained at a current density of 5 C with 0.1 M MnSO_4_


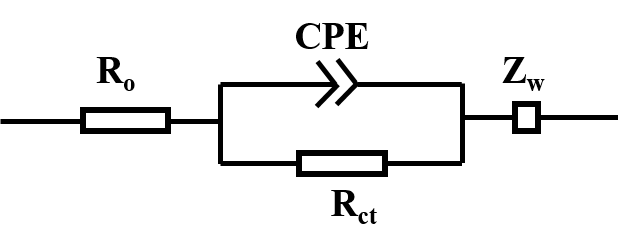


**Figure S8.** The equivalent circuit model of Zn-MnO_2_ micro battery used for fitting EIS.


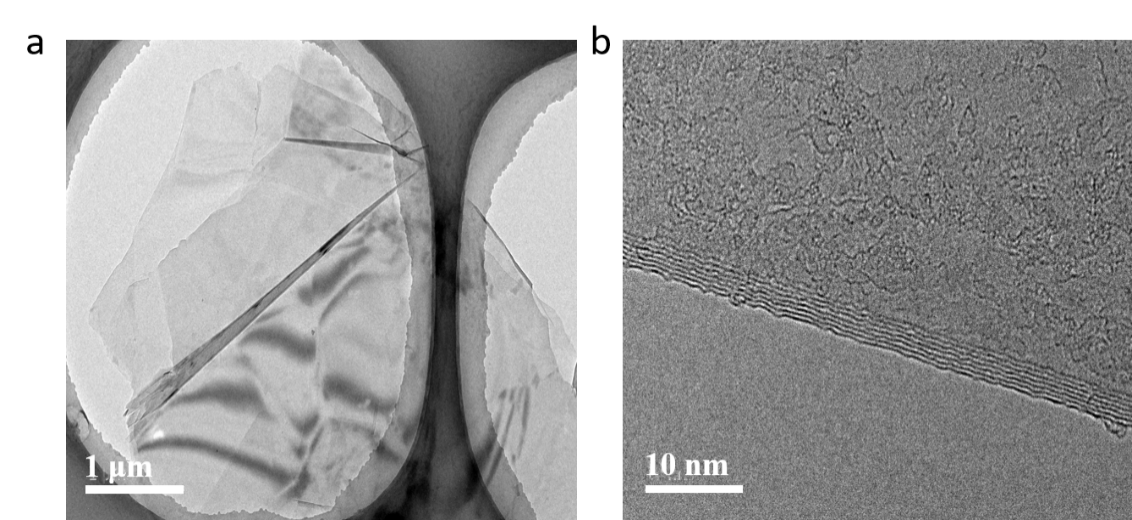


**Figure S9.** (a) TEM and (b) HRTEM images of graphene nanosheets.


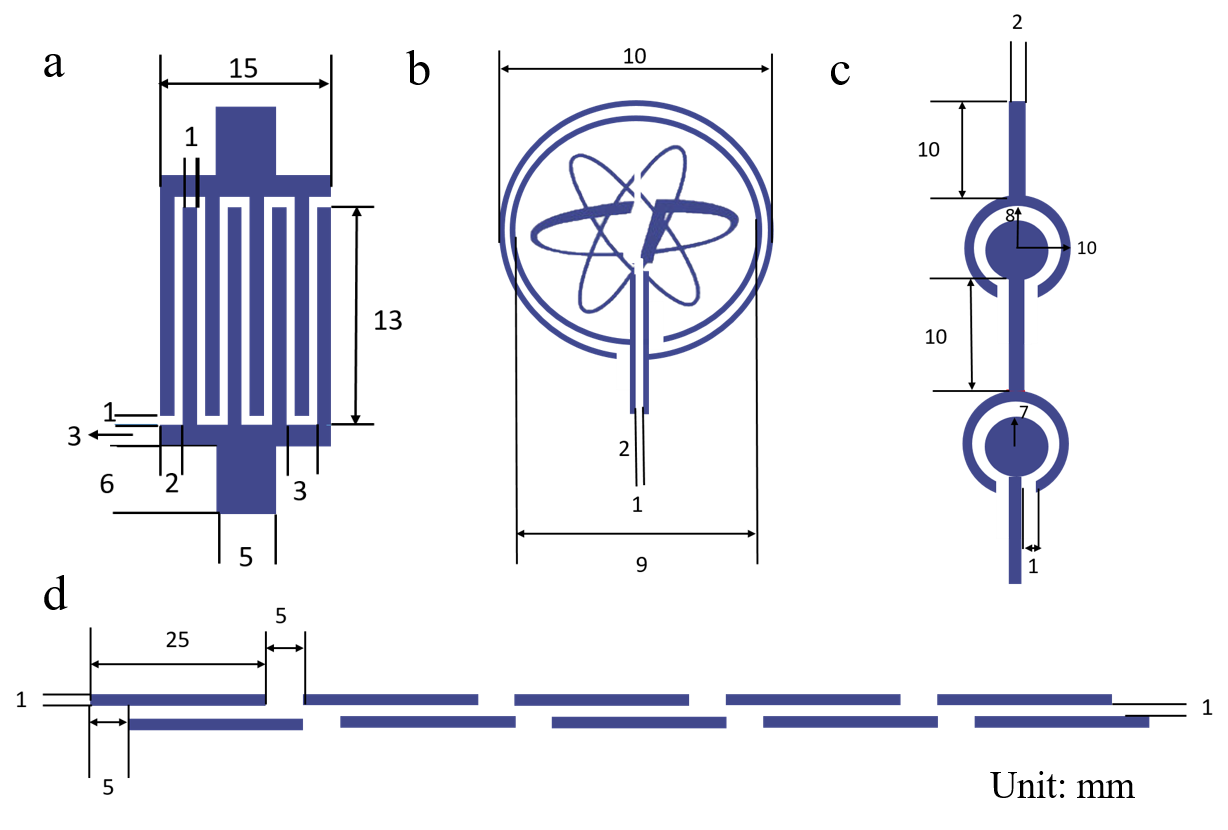


**Figure S10.** Microelectrode size parameters of shape-designable Zn//MnO_2_ MBs, including (a) interdigital, (b) our institute logo “DICP”, (c) concentric, and (d) linear shapes.


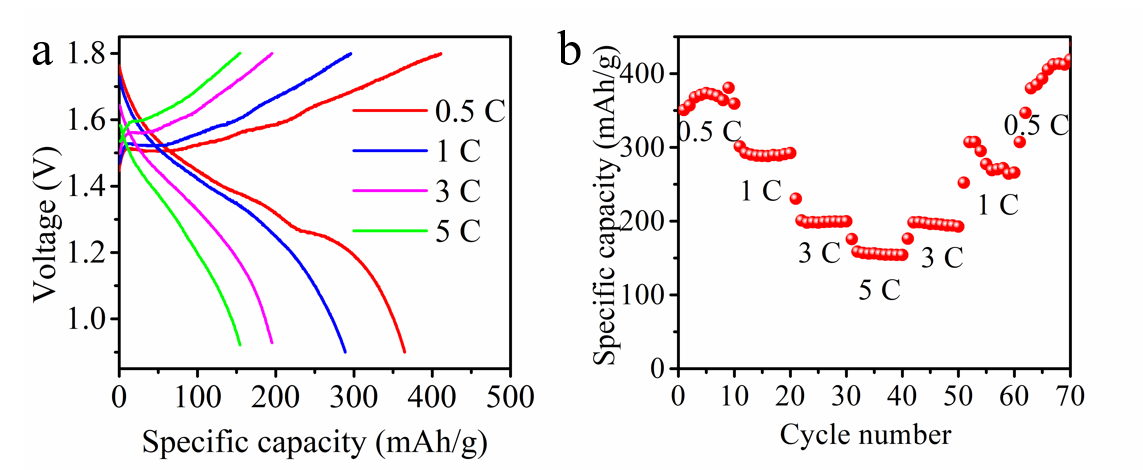


**Figure S11. (**a) The GCD profiles obtained from 0.5 C to 5 C, and (b) rate capability of planar Zn//MnO_2_ MBs.

**Table S1:** Performance comparison of Zn//MnO_2_ batteries with different electrolytes

| **Electrolyte** | **Capacity** | **Capacity retention** | **References** |
| --- | --- | --- | --- |
| Zn-alginate/PAAm hydrogel electrolyte | 300 mAh/g at 0.11 A/g | 82% after 500 cycles | [5] |
| EG-waPUA/PAM hydrogel electrolyte. | 146 mAh/g at 2.4 A/g | 88.36% after 600 cycles | [6] |
| 1M ZnSO_4_ or 1M Zn(NO_3_)_2_ | 210 mAh/g at 0.5C | 77% after 100 cycles | [7] |
| 1M ZnSO_4_ | 233mAh/g at 83 mA/g | 65% after 50 cycles | [8] |
| PVA/ZnCl_2_/MnSO_4_ | 367 mAh/g  at 0.74 A/g | 83.7% after 300 cycles | [9] |
| ZnSO_4_, MnSO_4_ Aqueous electrolyte | 393 mAh/g at 0.5 C | 83.9% after 1300 cycles | This work |

PAAm: polyacrylamide; EG: ethylene glycol; waPUA: waterborne anionic polyurethane acrylates; PAM: polyacrylamide; PVA: poly(vinyl alcohol).

**Table S2:** Performance comparison of our Zn//MnO_2_ MBs with traditional Zn//MnO_2_ batteries prepared by different methods

| Methods | Capacity | Capacity retention | Rate  capability | References |
| --- | --- | --- | --- | --- |
| Electrodeposition | 367 mAh/g  at 0.74 A/g | 83.7% after 300 cycles | 39% at 7.4 A/g | [9] |
| Electrodeposition | 5.35 mAh/cm^3^ at 1 C | 81.5% after 1000 cycles | 19% at 5 C | [10] |
| Manual processing | 169 mAh/g at 0.1 C | 90% after 300 cycles | 58% at 3 C | [11] |
| Screen printing | 393 mAh/g at 0.5 C | 83.9% after 1300 cycles | 42% at 5 C | This work |

**References**

1. Zeng, Y. X., Zhang, X. Y., and Meng, Y.*, et.al.* Achieving ultrahigh energy density and long durability in a flexible rechargeable quasi-solid-state Zn-MnO_2_ battery. *Adv. Mater.* 2017; **29**: 1700274.
2. Fu, Y., Wei, Q., and Zhang, G.*, et.al.* High-performance reversible aqueous zn-ion battery based on porous MnO_x_ nanorods coated by MOF derived N-doped carbon. *Adv. Energy Mater.* 2018; **8:** 1801445.
3. Wang, Z. Q., Winslow, R., and Madan, D.*, et.al.* Development of MnO_2_ cathode inks for flexographically printed rechargeable zinc-based battery. *J. Power Sources* 2014; **268**: 246-54.
4. Gaikwad, A. M., Whiting, G. L., and Steingart, D. A.*, et.al.* Highly flexible, printed alkaline batteries based on mesh-embedded electrodes. *Adv. Mater.* 2011; **23**: 3251-55.
5. Liu, Z., Wang, D., and Tang, Z., *et.al.* A mechanically durable and device-level tough Zn-MnO_2_ battery with high flexibility. *Energy Storage Mater.* 2019, DOI: 10.1016/j.ensm.2019.03.007.
6. Mo, F., Liang, G., and Meng, Q., *et.al.* A flexible rechargeable aqueous zinc manganese-dioxide battery working at −20℃. *Energ. Environ. Sci.* 2019; **12**: 706-15.
7. Xu, C., Li, B., and Du, H., *et.al.* Energetic zinc ion chemistry: The rechargeable zinc ion battery. *Angew. Chem. Int. Ed.* 2012; **51**: 933-5.
8. Alfaruqi, M. H., Gim, J., and Kim, S., *et.al*. Enhanced reversible divalent zinc storage in a structurally stable α-MnO_2_ nanorod electrode. *J. Power Sources* 2015; **288**: 320-27.
9. Zeng, Y. X., Zhang, X. Y., and Meng, Y., *et.al.* Achieving ultrahigh energy density and long durability in a flexible rechargeable quasi-solid-state Zn-MnO_2_ battery. *Adv. Mater.* 2017; **29**: 1700274
10. Lai, W. H., Wang, Y., Lei, and Z. W., *et.al*. High performance, environmentally benign and integratable Zn//MnO2 microbatteries. *J. Mater. Chem. A* 2018; **6**: 3933-40.
11. Zhao, J. W., Sonigara, K. K., and Li, J. J., *et.al*. A smart flexible zinc battery with cooling recovery ability. *Angew. Chem. Int. Ed.* 2017; **56**: 7871-75.
